# Characterization of Novel CSF Tau and ptau Biomarkers for Alzheimer’s Disease

**DOI:** 10.1371/journal.pone.0076523

**Published:** 2013-10-07

**Authors:** Jere E. Meredith Jr., Sethu Sankaranarayanan, Valerie Guss, Anthony J. Lanzetti, Flora Berisha, Robert J. Neely, J. Randall Slemmon, Erik Portelius, Henrik Zetterberg, Kaj Blennow, Holly Soares, Michael Ahlijanian, Charles F. Albright

**Affiliations:** 1 Research and Development, Bristol-Myers Squibb, Wallingford, Connecticut, United States of America; 2 Research and Development, Bristol-Myers Squibb, Lawrenceville, New Jersey, United States of America; 3 Institute of Neuroscience and Physiology, Department of Psychiatry and Neurochemistry, The Sahlgrenska Academy, University of Gothenburg, Mölndal, Sweden; 4 UCL Institute of Neurology, London, United Kingdom; National Center for Geriatrics and Gerontology, Japan

## Abstract

Cerebral spinal fluid (CSF) Aβ42, tau and p181tau are widely accepted biomarkers of Alzheimer’s disease (AD). Numerous studies show that CSF tau and p181tau levels are elevated in mild-to-moderate AD compared to age-matched controls. In addition, these increases might predict preclinical AD in cognitively normal elderly. Despite their importance as biomarkers, the molecular nature of CSF tau and ptau is not known. In the current study, reverse-phase high performance liquid chromatography was used to enrich and concentrate tau prior to western-blot analysis. Multiple N-terminal and mid-domain fragments of tau were detected in pooled CSF with apparent sizes ranging from <20 kDa to ~40 kDa. The pattern of tau fragments in AD and control samples were similar. In contrast, full-length tau and C-terminal-containing fragments were not detected. To quantify levels, five tau ELISAs and three ptau ELISAs were developed to detect different overlapping regions of the protein. The discriminatory potential of each assay was determined using 20 AD and 20 age-matched control CSF samples. Of the tau ELISAs, the two assays specific for tau containing N-terminal sequences, amino acids 9-198 (numbering based on tau 441) and 9-163, exhibited the most significant differences between AD and control samples. In contrast, CSF tau was not detected with an ELISA specific for a more C-terminal region (amino acids 159-335). Significant discrimination was also observed with ptau assays measuring amino acids 159-p181 and 159-p231. Interestingly, the discriminatory potential of p181 was reduced when measured in the context of tau species containing amino acids 9-p181. Taken together, these results demonstrate that tau in CSF occurs as a series of fragments and that discrimination of AD from control is dependent on the subset of tau species measured. These assays provide novel tools to investigate CSF tau and ptau as biomarkers for other neurodegenerative diseases.

## Introduction

Alzheimer’s disease (AD) is the most common form of dementia and the sixth leading cause of death in the US. Estimates indicate that ~5 million people in the US currently have the disease and that number is expected to increase up to ~16 million by 2050. Current therapeutic options are limited to symptomatic treatments highlighting the urgent need to develop and evaluate novel disease-modifying approaches. Identification of sensitive and specific AD biomarkers will be critical for the development of these therapeutics.

Over the past two decades, extensive effort has focused on the identification and development of AD biomarkers specifically linked to disease pathology (reviewed in 1). The major histopathological features of AD are senile plaques and neurofibrillary tangles (NFT) (reviewed in 2). Senile plaques are comprised of extracellular deposits of Aβ peptides, primarily Aβ42, which are generated by proteolytic processing of the amyloid precursor protein. NFTs are formed from the aggregation of hyperphosphorylated tau, a protein normally associated with microtubules. Numerous studies show that cerebral spinal fluid (CSF) Aβ42 levels decrease to around half the level in controls while CSF tau and p181tau levels increase around 2-3 fold in mild-moderate AD patients compared to age-matched controls (e.g. [3,4,5]). In addition to p181tau, measures of other phospho-epitopes, including p199, p212/p214, p231, p231/p235, p396/p404, have also been reported to be increased in AD relative to age-matched controls [6,7,8,9,10,11]. Changes in Aβ42, tau and p181tau are evident many years prior to onset of dementia and are predictive of conversion to mild AD [3,4,12,13,14,15].

The decrease in CSF Aβ42 observed in AD patients is thought to reflect increased binding and sequestration of Aβ42 in senile plaques present in the diseased brain [16,17,18,19,20]. In contrast, multiple mechanisms have been proposed to explain increased CSF tau and ptau in AD. For example, CSF tau levels in stroke, traumatic brain injury and Creutzfeldt–Jakob disease increase rapidly and dramatically likely due to acute neuronal cell death [21,22,23,24]. Interestingly, these increases in CSF tau are not associated with any change in CSF pTau (e.g. [24,25]), suggesting that CSF pTau is not a general marker for neuronal damage or degeneration. Neuron cell loss is also a hallmark of AD and thus could explain some of the increases in CSF tau; however, cell loss develops relatively slowly in AD and thus is unlikely to be the only cause. Moreover, increases in CSF tau are not detected in other neurodegenerative diseases despite ongoing neuronal cell loss (e.g. PD, FTD). Recent evidence from in vitro studies indicates that tau can be actively secreted from cells [26,27,28,29,30,31]. Such a secretion process could help explain the presence of CSF tau and ptau in normal healthy subjects. Thus the increase in CSF tau and ptau observed in AD likely reflects a combination of neuronal cell death and active secretion.

The molecular nature of tau in CSF is also unknown. Various reports suggest that fragments of tau are present in CSF, though the exact identity of these fragments is not defined [7,22,27,32,33,34,35,36,37]. The majority of CSF tau and ptau data reported in the literature is based on two related commercially available assays, INNO-BIA AlzBio3 and the INNOTEST plate ELISAs [38,39]. In these assays, total tau and ptau measurements are dependent on anti-tau antibodies (AT120, HT7 and BT2) specific for the mid-domain region of the protein (Table 1). The potentially limited range of tau species measured by these assays, given evidence for the presence of tau fragments, raises concerns regarding the effect this could have on diagnostic accuracy and outcome measures. Additional tools to enable a more comprehensive analysis of tau and ptau species in CSF are clearly needed.

**Table 1:** Tau and ptau antibodies.

**Clone**	**Vendor**	**Cat #**	**Epitope^1^**	**Species**	**Reference**
**BT2**	Thermo Scientific	MN1010	194-198	Mouse	[38,49]
**HT7**	Thermo Scientific	MN1000	159-163	Mouse	[38]
**Tau5**	Covance	SIG-39413	218-225	Mouse	[52,53]
**Tau12**	Covance	SIG-39416	9-18	Mouse	[54]
**KJ9A**	Dako	A 0024	243-441	Rabbit	
**77G7**	Covance	SIG-39405	316-335^2^	Mouse	this paper
**PHF6**	Covance	SIG-39430	pT231	Mouse	[55]
**AT270**	Thermo Scientific	MN1050	pT181	Mouse	[56]
**IgG**	Abcam	ab81032	NA	Mouse	NA

1 Amino acid numbering based on human tau 441 sequence

**Figure 1:**
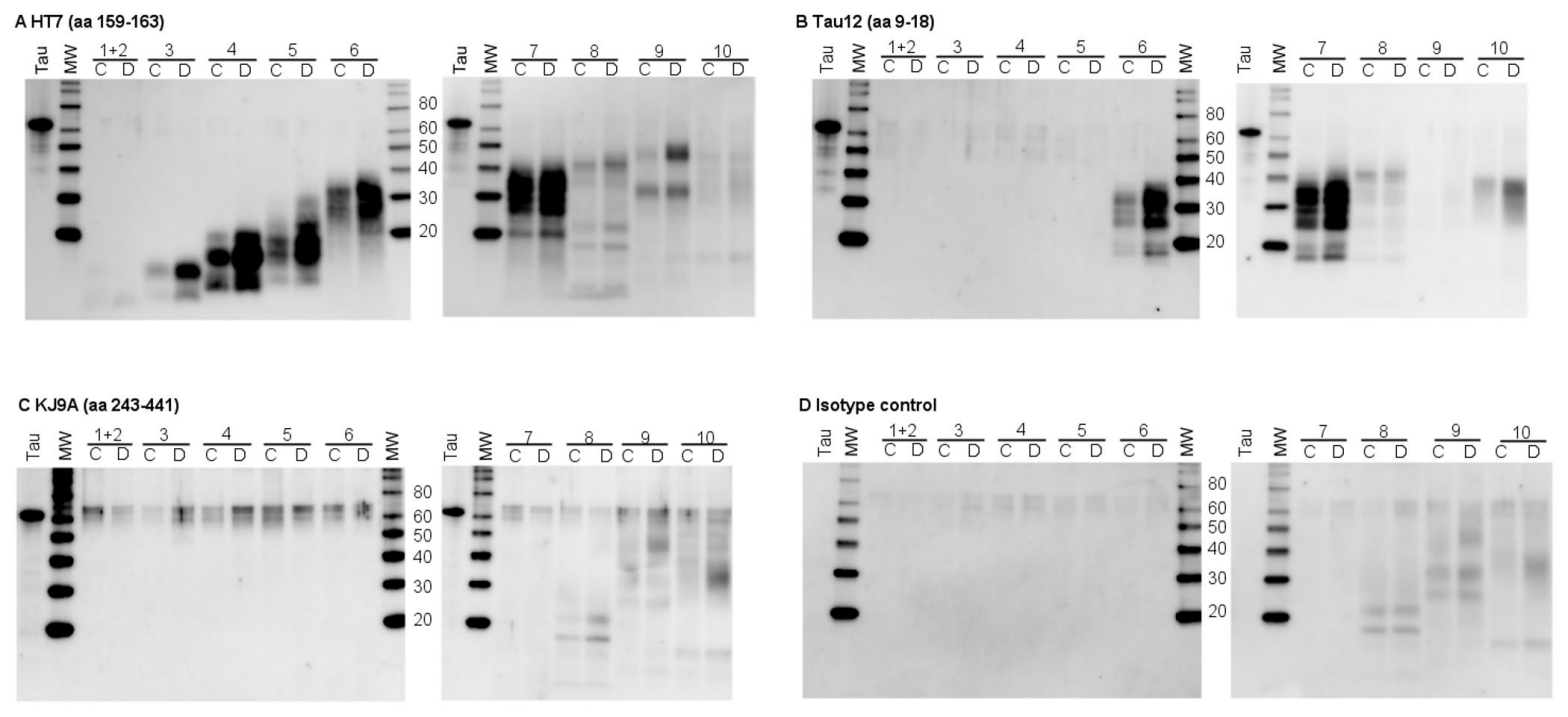
Detection of tau fragments in human CSF. Human control and AD CSF subjected to RP-HPLC, fractions collected and run on SDS-PAGE gels followed by western-blotting with different tau antibodies. A) HT7 (mid domain antibody). B) Tau 12 (N-terminal antibody). C) K9JA (C-terminal microtubule repeat domain antibody). D) IgG1 isotype control. On each blot, human recombinant tau441 (tau) is included in lane 1 and molecular weight markers (mw) in lane 2 followed by the HPLC fractions from 1 to 6 or HPLC fractions 7 to 11. Fractions 1 and 2 were pooled and run as a single sample, while fractions 3-10 were run as individual samples. Control CSF (C) and AD CSF (D) samples for each fraction were run side by side for comparison.

**Figure 2:**
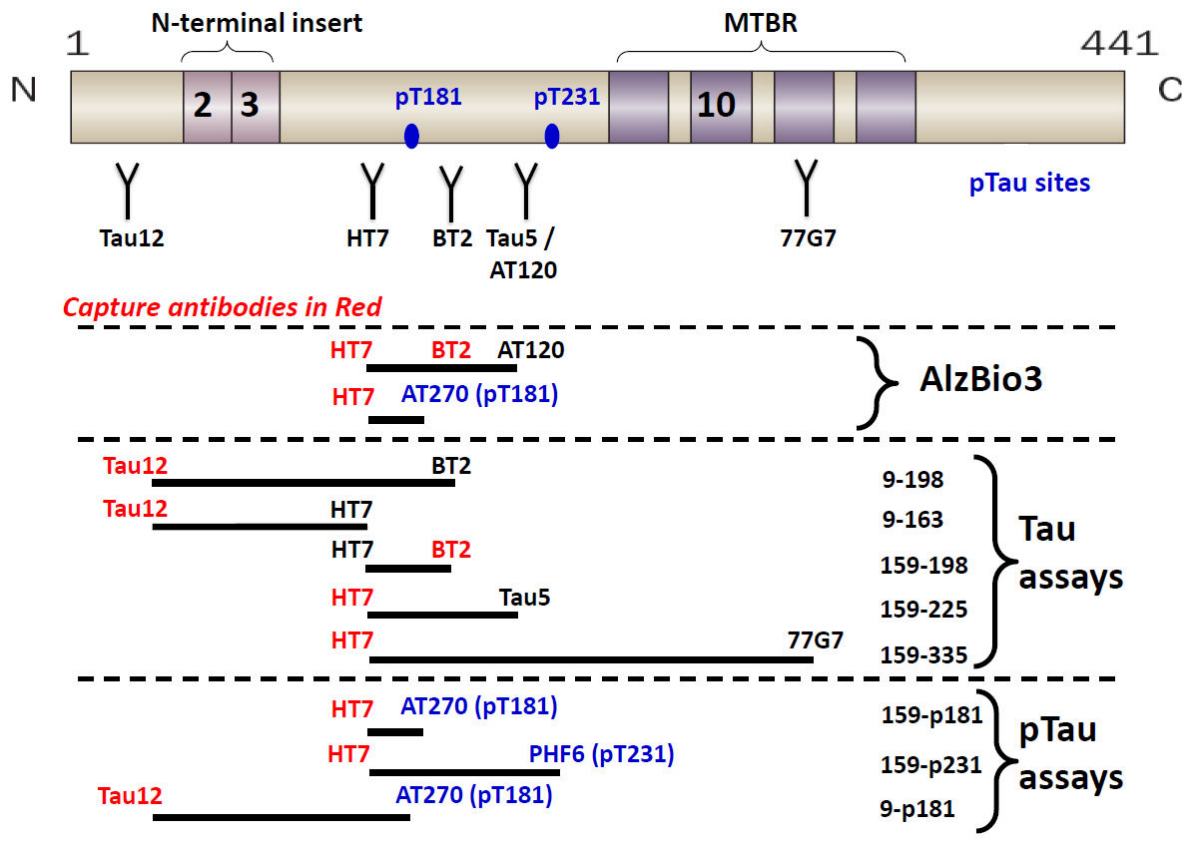
Tau and ptau ELISAs. Schematic of tau 441 protein with the approximate location of various linear epitope antibodies Tau12, HT7, BT2, Tau5, AT120 and 77G7 and phospho-site specific antibodies AT270 (p181) and PHF6 (p231) indicated; additional antibody epitope and clone information included in Table 1. Antibody combinations used for the different tau and ptau ELISAs are shown. For each assay, capture antibodies are highlighted in red, detection antibodies in black and the minimal tau region required (aa numbering based on tau 441) is indicated. Antibodies used in the INNOTEST/INNO-BIA AlzBio3 total tau and p181 tau assays are also shown for comparison.

2 Epitope mapping included in Supplemental methods and Figure 1 and Figure 2.

NA: not applicable

In this study, we developed a sensitive western-blotting method to characterize the tau profile in CSF. In addition, we developed a set of novel overlapping tau and ptau ELISAs to measure different tau and ptau species and to evaluate the ability of these to discriminate between AD and control CSF samples. Results from the western-blotting analysis demonstrate that CSF tau is composed of a series of fragments while results from the ELISAs suggest that discriminatory potential of tau and ptau is dependent on the particular species measured.

## Results

### Tau fragments detected in human CSF by western-blotting

To determine the nature of tau in human CSF, a reverse-phase high performance liquid chromatography column (RP-HPLC) was used to enrich the relatively low abundance tau protein prior to western-blot analysis. Equal volumes of pooled control and AD CSF samples were fractionated and then western-blots analyzed with antibodies specific for different regions of the protein; a summary of the antibodies used is shown in Table 1. A range of bands were detected with HT7, an antibody specific for a mid-domain epitope (amino acids (aa) 159-163; amino acid numbering based on human tau 441) (Figure 1A). The majority of bands present in fractions 3 to 7 were specific for HT7, as these were not detected with the IgG1 isotype control antibody (Figure 1D). These bands exhibited a range of apparent molecular weights (MWs), from < 20 kDa to ~40 kDa, suggesting the presence of tau protein fragments. Interestingly, tau-specific bands that comigrated with the tau441 standard (~65 kDa) were not detected suggesting that full length tau is not present. A subset of the HT7-immunoreactive bands in fractions 6 to 8 was also detected with the N-terminal antibody Tau12 (epitope aa 9-18) indicating that these fragments span both N-terminal and mid-domain regions of tau (Figure 1B). In addition, bands only detected with Tau12 were also observed in fractions 6 and 7 providing evidence that truncated N-terminal tau fragments are also present. Consistent with the HT7 findings, bands of ~65 kDa were not detected with Tau12, confirming the lack of full-length tau in CSF. Interestingly, the overall tau fragment pattern observed with both HT7 and Tau12 was similar in AD compared to control.

Fractions were also analyzed for the presence of C-terminal fragments using the rabbit polyclonal antibody K9JA, which binds to the microtubule repeat and C-terminal flanking region of tau (aa 243-441). K9JA exhibited robust staining of full length tau441 standard (Figure 1C); however, unlike HT7 and Tau12, K9JA-specific tau bands were not detected in the CSF samples (Figure 1C). Similar results were also observed with the MTBR-specific antibody 77G7 (data not shown). A set of bands of 60-65 kDa were detected at various levels in all of the fractions (Figure 1C), though these are likely due to nonspecific immunoreactivity as they were detected to a lesser degree with the isotype control antibody (Figure 1D), and appear to be due to cross-reactivity with contaminating keratin (data not shown); the lack of full length tau is also consistent with ELISA data discussed below. Taken together, these results indicate that tau in both control and AD CSF is present as a set of primarily N-terminal and mid-domain fragments.

### Tau fragment and ptau assays

To more accurately quantify CSF tau levels, a set of novel tau and ptau ELISAs were developed (Figure 2). Assays were designed to measure overlapping regions of tau using different combinations of tau and ptau antibodies (Table 1). Each assay requires different minimal regions of tau, as defined by the epitopes of the antibodies used, and thus will measure tau fragments containing these regions. The minimal regions of the tau assays are aa 9-163 (Tau12-HT7), aa 9-198 (Tau12-BT2), aa 159-198 (HT7-BT2), aa 159-225 (HT7-Tau5), aa 159-335 (HT7-77G7); the minimal regions of the ptau assays are aa 9-p181 (Tau12-AT270), aa 159-p181 (HT7-AT270) and aa 159-p231 (HT7-PHF6) (Figure 2).

**Figure 3:**
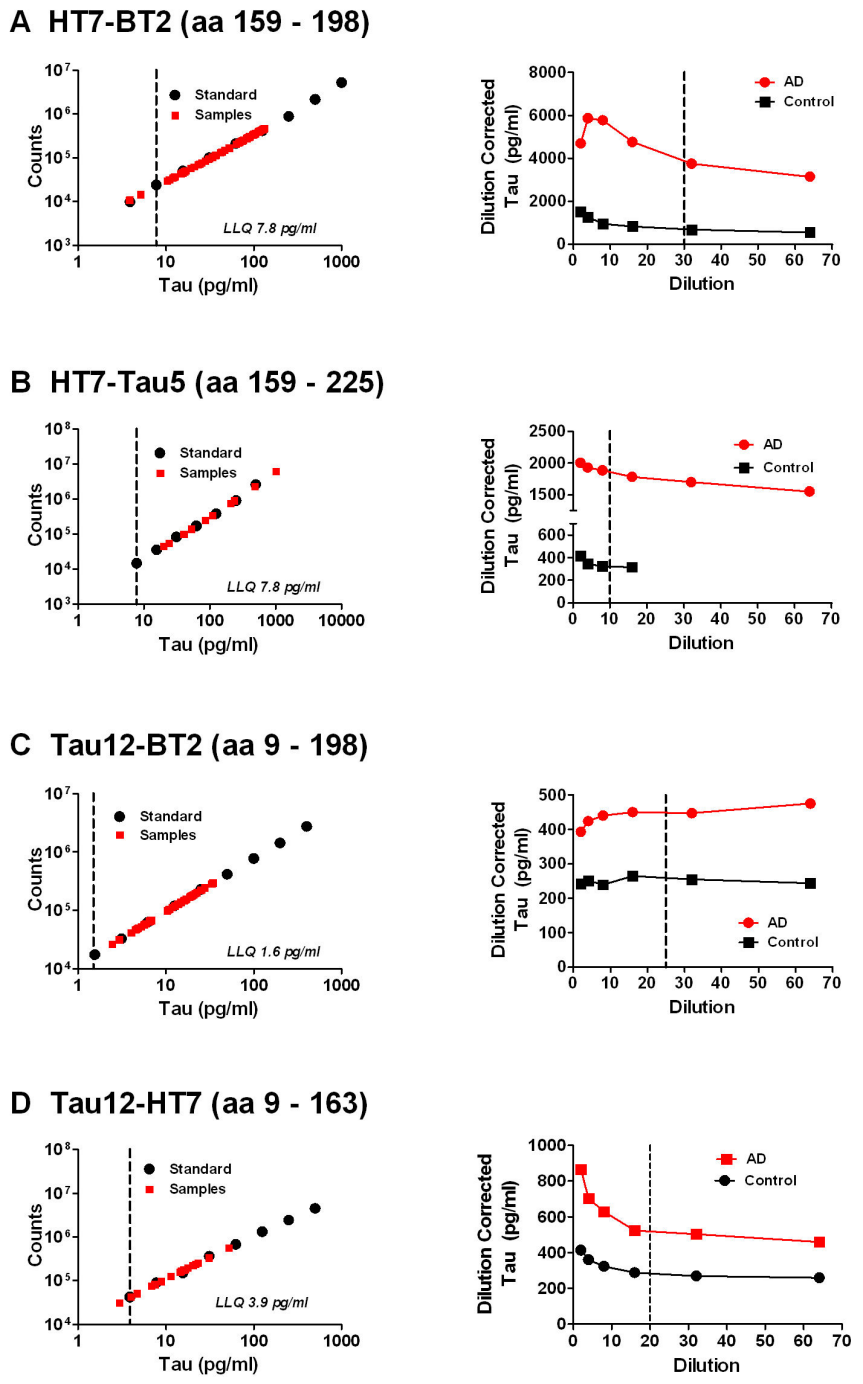
Characterization of tau ELISAs. Representative tau 441 standard curves (left panels) and CSF dilution linearity results (right panels) shown for A) HT7-BT2, B) HT7-Tau5, C) Tau12-BT2 and D) Tau12-HT7 tau ELISAs. On each standard curve graph, tau 441 calibrators (Standards) and results for CSF sample dilutions (Samples) are shown. The assay lower limit of quantitation (LLQ, vertical dashed line) is also indicated. On each dilution linearity graph, dilution-corrected tau levels for a pooled control CSF sample and a pooled AD CSF sample relative to sample dilution are shown. The vertical dashed lines indicate the dilution determined to be optimal for CSF analysis.

The HT7-BT2, HT7-Tau5, Tau12-BT2 and Tau12-HT7 tau assays demonstrated ~100-fold dynamic range of quantitation using tau 441 standard, with the LLQ ranging from 1.6 pg/ml to 7.8 pg/ml (Figure 3). Signal from pooled AD and control CSF samples were within the dynamic range of the HT7-BT2 assay when diluted 2- to 64-fold (left panel, Figure 3A). Consistent dilution-corrected tau levels were observed with CSF dilutions ranging from 16- to 64-fold; similar results were observed for both AD and control samples (right panel, Figure 3A). A dilution of 30-fold was identified as optimal for CSF sample analysis. Similar results were observed for HT7-Tau5, Tau12-BT2 and Tau12-HT7 (Figure 3B, C, D, respectively) with optimal CSF dilutions of 10-fold, 25-fold and 20-fold, respectively. Tau specificity in each assay was verified based on immuno-depletion (Figure S3) and spike recovery (Figure S4). Taken together, these results confirm the ability of these assays to accurately measure tau in CSF.

**Figure 4:**
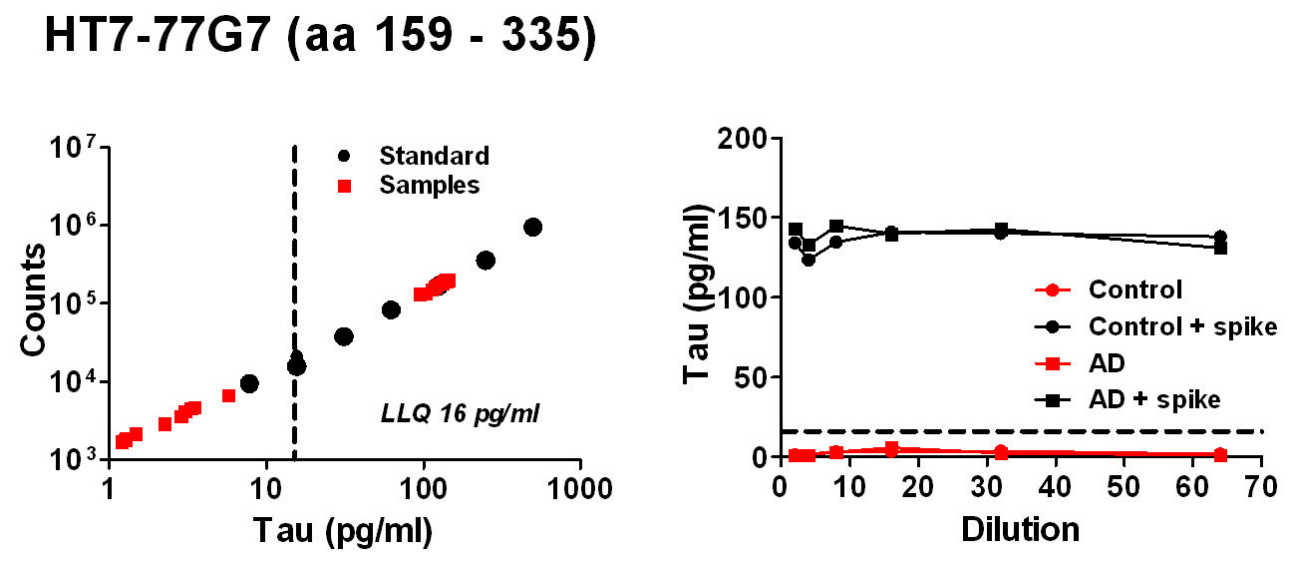
Characterization of HT7+77G7 tau ELISA. Representative tau 441 standard curve (left panel) and CSF dilution linearity results (right panel) shown. On the standard curve graph, tau 441 calibrators (Standards) and results for CSF sample dilutions (Samples) are shown. The assay lower limit of quantitation (LLQ, vertical dashed line) is also indicated. On the dilution linearity graph, tau levels for a pooled control and pooled AD CSF samples tested with or without a 100 pg/ml tau 441 spike are shown.

The HT7-77G7 assay is specific for tau species containing more C-terminal sequences (aa 159-335). The dynamic range and LLQ observed (16 pg/ml) were similar to the other tau ELISAs (Figure 4); however, a HT7-77G7 signal was not detected in either the pooled control or pooled AD samples, regardless of sample dilution (Figure 4). The lack of signal was not an artifact of matrix interference as robust recovery of a 100 pg/ml tau 441 spike was observed in both control and AD CSF over a range of dilutions (Figure 4). These results indicate that tau species containing the region aa 159-335 are not present in these pooled CSF samples at levels >16 pg/ml.

**Figure 5:**
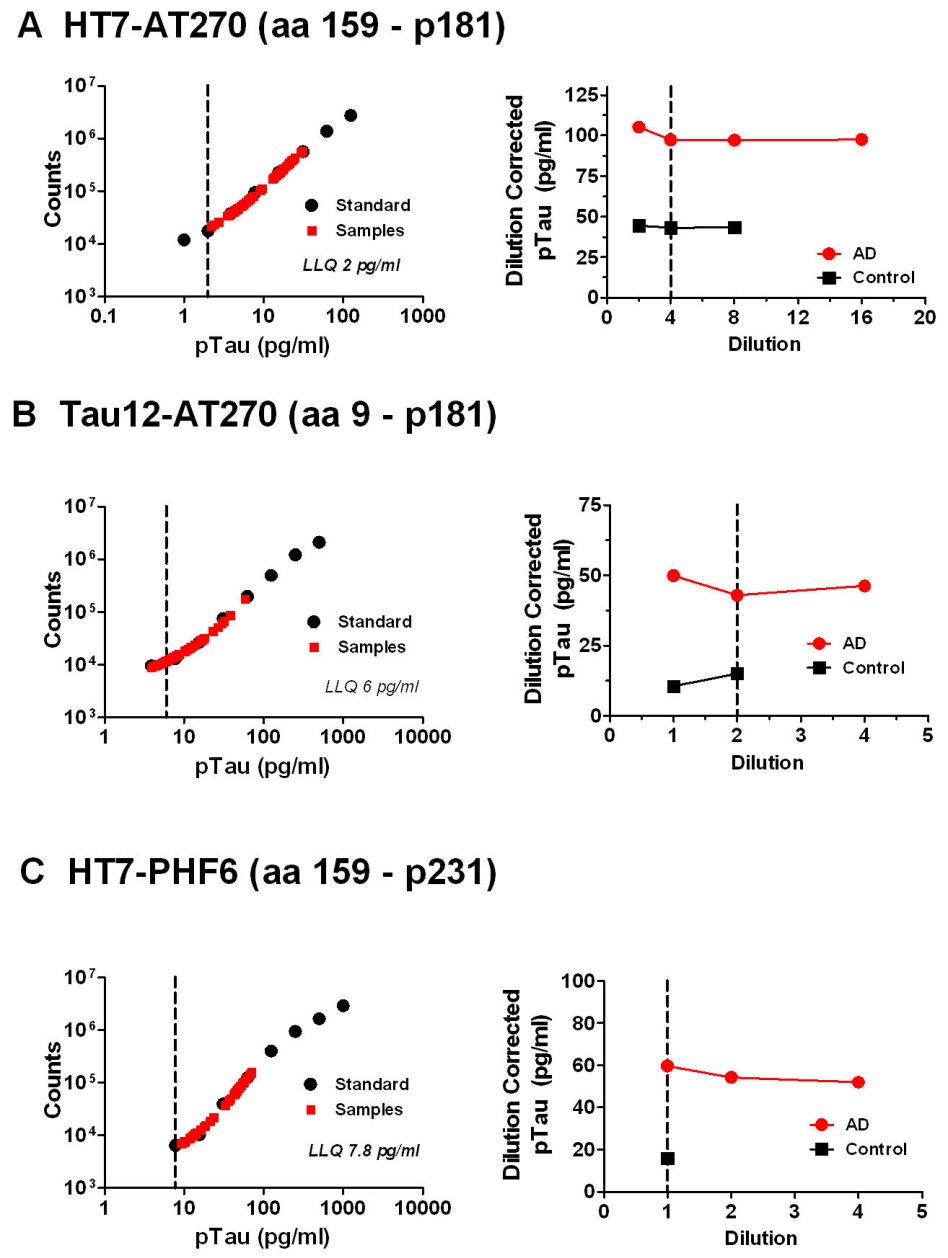
Characterization of ptau assays. Representative ptau standard curves (left panels) and CSF dilution linearity results (right panels) shown for A) HT7-AT270, B) HT7-PHF6, and C) Tau12-AT270 ptau ELISAs. On each standard curve graph, ptau calibrators (Standards) and results for CSF sample dilutions (Samples) are shown. The assay lower limit of quantitation (LLQ, vertical dashed line) is also indicated. On each dilution linearity graph, dilution-corrected ptau levels for a pooled control and a pooled AD CSF samples relative to sample dilution are shown. The vertical dashed lines indicate the dilution determined to be optimal for CSF analysis.

All three ptau assays, HT7-AT270, Tau12-AT270 and HT7-PHF6, demonstrated ~100-fold dynamic range of quantitation using synthetic ptau standards, with LLQs ranging from 2 pg/ml to 7.8 pg/ml (Figure 5). In the HT7-AT270 assay, consistent dilution-corrected ptau levels were observed in AD and control CSF with dilutions ranging from 2- to 16-fold (right panel, Figure 5A). In the Tau12-AT270 assay (Figure 5B) and HT7-PHF6 assays (Figure 5C), dilution linearity was observed when samples were measured neat or diluted up to 4-fold. CSF ptau signal specificity was verified by a combination of immuno-depletion and peptide competition (Figure S5) and spike recovery (Figure S6). Taken together, these results confirm specificity of the ptau signal measured in these assays.

### Evaluation of CSF tau and ptau in a cohort of control and AD CSF samples

To evaluate the discriminatory power of the assays, tau and ptau levels were measured in a cohort of 20 AD and 20 age-matched control CSF samples (20x20 sample set); demographic information included in Table 2. The relative ability of each assay to detect differences between AD and control samples was assessed using Student’s t-test comparison of log-transformed data. Differences were deemed significant for p < 0.01. Samples were also benchmarked for Aβ42, tau and p181tau using INNO-BIA AlzBio3. Levels of CSF Aβ42 were significantly reduced while levels of tau and ptau were significantly increased in AD samples compared to the age-matched controls when measured using INNO-BIA AlzBio3 (Figure S7; Table 3), consistent with expectations for a typical AD vs control sample set.

**Table 2:** Demographics of 20x20 CSF sample set.

	**Control**	**AD**
**n**	20	20
**Age at LP (SD), yr**	68 (6)	72 (6)
**Gender F/M**	10/10	10/10
**MMSE (SD)**	30 (0.5)	21 (4)

**Table 3:** Summary of analysis of 20x20 CSF sample set.

			**Raw data, pg/ml^1^**		**Log transformed data^1^**			
**Assay**			**Control**	**AD**	**Control**	**AD**	**Fold-diff**	**p-value^2^**
**AlzBio 3**	**Aβ42**		262 (57)	184 (78)	2.409 (0.096)	2.225 (0.192)	0.65	0.0005
	**Tau**		48 (17)	99 (66)	1.662 (0.143)	1.920 (0.257)	1.8	0.0003
	**pTau**		23 (7)	46 (28)	1.330 (0.147)	1.591 (0.249)	1.8	0.0003
**Tau**	**Tau12-HT7**	**(aa 9-163)**	312 (95)	714 (497)	2.474 (0.137)	2.773 (0.267)	2.0	<0.0001
	**Tau12-BT2**	**(aa 9-198)**	591 (194)	1162 (639)	2.747 (0.155)	3.011 (0.223)	1.8	0.0001
	**HT7-BT2**	**(aa 159-198)**	1556 (563)	2546 (1914)	3.112 (0.153)	3.330 (0.268)	1.7	0.0031
	**HT7-Tau5**	**(aa 159-225)**	379 (185)	1019 (888)	2.630 (0.159)	2.943 (0.308)	2.1	0.0003
	**HT7-77G7**	**(aa 159-335)**	<LLQ	<LLQ				
**pTau**	**HT7-AT270**	**(aa 159-p181)**	46 (15)	81 (44)	1.646 (0.132)	1.856 (0.208)	1.6	0.0005
	**HT7-PHF6**	**(aa 159-p231)**	18 (18)	43 (28)	1.122 (0.331)	1.501 (0.382)	2.4	0.0018
	**Tau12-AT270**	**(aa 9 -p181)**	21 (7)	29 (10)	1.306 (0.162)	1.439 (0.148)	1.4	0.0100

1 Data based on n = 20 control, n = 20 AD samples. Values represent mean (SD)

2 p-values based on unpaired, 2-tailed Student’s t test comparison of log transformed control and AD data

LLQ: Lower limit of quantitation

**Figure 6:**
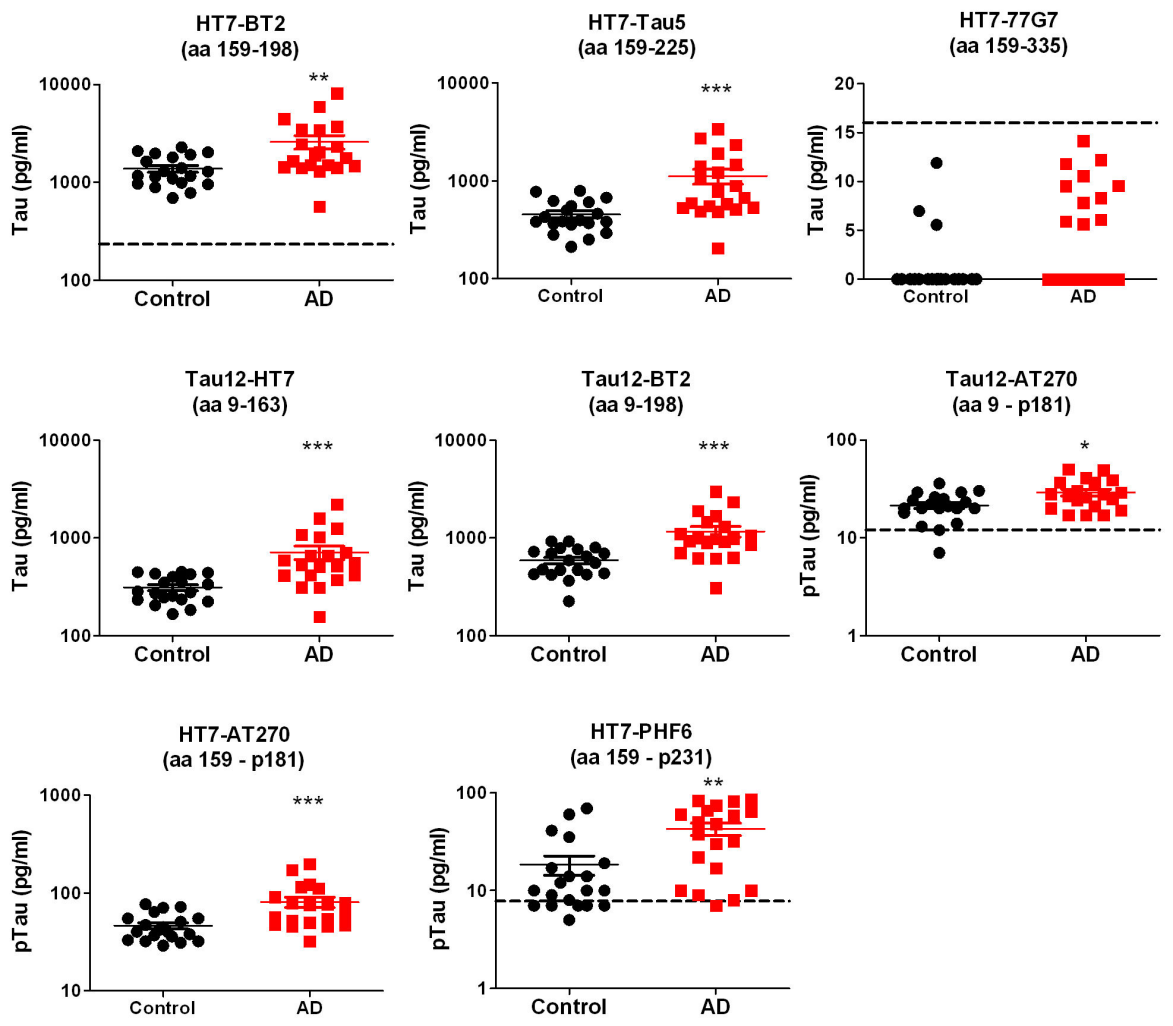
Tau and ptau levels in 20 AD and 20 control CSF samples. A set of 20 AD and 20 age-matched normal control CSF samples were analyzed using the tau ELISAs (HT7-BT2, HT7-Tau5, Tau12-BT2, Tau12-HT7 and HT7-77G7) and pTau ELISAs (HT7-AT270, HT7-PHF6 and Tau12-AT270). Dashed lines indicate the assay LLQ corrected for CSF dilution. Statistics based on 2-tailed Student’s t test comparison of log-transformed data, * p < 0.05; ** p< 0.01; *** p < 0.001.

In the tau ELISAs, the highest CSF tau levels were detected using the HT7-BT2 assay, specific for tau species containing aa 159-198. In comparison, levels of tau species containing additional N-terminal sequence (aa 9-198, Tau12-BT2; aa 9-163, Tau12-HT7) were 2- to 4-fold lower, while tau species containing additional C-terminal sequence (aa 159-225, HT7-Tau5) were 3-fold lower (Table S1). Levels measured in these assays were highly correlated (r^2^ = 0.87-0.95) (Table S2). On the other hand, tau containing additional C-terminal sequence aa 159-335 (HT7-77G7) could not be detected (Figure 6), consistent with results from the pooled samples (Figure 4). A signal above background was detected in 11 of the 20 AD samples and 3 of the 20 control samples though additional work will be needed to quantify and verify the specificity of this HT7-77G7 signal.

Overall, CSF tau levels were found to be significantly higher in AD samples compared to controls in all but the HT7-77G7 assay (Figure 6; Table 3). The most significant differences were observed using assays specific for tau containing aa 9-163 (Tau12-HT7) and aa 9-198 (Tau12-BT2), compared to the difference detected with the HT7-BT2 assay, specific for tau species containing aa 159-198. Taken together, these results indicate that the discriminatory power of CSF tau is dependent on the tau species measured.

The 20x20 samples were also analyzed using the ptau ELISAs. All three ptau ELISAs exhibited significantly higher levels in AD compared to control samples, with increases ranging from 1.4-fold to 2.4-fold (Figure 6; Table 3). Of these, HT7-AT270, specific for tau species containing aa 159-p181, exhibited the most significant difference between AD and control samples. Interestingly, the level of significance was reduced when p181 was measured in the context of tau species containing additional N-terminal sequence aa 9-p181 (Tau12-AT270) (p = 0.01, Table 3). Thus, similar to results for the tau ELISAs, the discriminatory power of CSF p181 is dependent on the tau species measured. Significant discrimination of AD from control samples was also observed using HT7-PHF6, specific for aa 159-p231, though less than observed with the HT7-AT270 assay. In comparison to the tau ELISA results, a weaker degree of correlation was observed using the ptau measures (Table S1). Of the 3 assays, HT7-AT270 demonstrated the highest degree of correlation with the tau assays (r^2^ =0.67-0.72). By comparison, the HT7-PHF6 and Tau12-AT270 correlations were less robust (r^2^ =0.18-0.37) and in some cases not significant.

To further compare the discriminatory power of the different tau and ptau assays, all possible combinations of tau and ptau ratios were calculated for each individual CSF sample and then these values used to evaluate differences and discrimination between AD and control samples (Table 3, Table S1, Table S3). This analysis of ratios enables comparison of the relative disease-associated changes between different assays. Of all the ratios analyzed, only two, HT7-BT2/HT7-Tau5 and HT7-BT2/Tau12-HT7, exhibited significant differences between AD and control (Table S1). In contrast, HT7-Tau5/Tau12-HT7 levels in AD and control samples were nearly identical (Table S1). These results further support the idea that discrimination of AD from controls is dependent on the subset of CSF tau species measured.

## Discussion

In this study, tau profiles and the relative differences in tau and ptau levels between AD and age-matched control CSF samples were investigated using a combination of qualitative and quantitative biochemical assays. A sensitive western-blotting method was used to demonstrate that tau is present in CSF as a series of N-terminal and mid-domain fragments. Results from a set of novel ELISAs specific for different overlapping regions of tau demonstrated that the ability of CSF tau and ptau to differentiate AD from control is dependent on the tau species measured. These endpoints provide novel tools to investigate CSF tau in AD and other neurodegenerative disorders.

A number of previous studies have reported on the characterization of tau in CSF using western-blotting-based techniques; however, results from these studies are mixed and incongruent [7,22,27,32,33,34,35,36,37,40,41]. Many studies report the presence of tau fragments in CSF, though the size, number and prevalence of these fragments vary. Many also report the presence of full-length tau and in two studies only full-length tau was detected [40,41]. Some of the discrepancies between studies may be due to nonspecific binding artifacts commonly observed with western-blotting. We have identified a number of nonspecific binding activities in fractionated samples with various tau-specific antibodies (data not shown). Indeed, such nonspecific artifacts may account for some of the findings reported ( [35,36]; see 42). In some studies, incorporation of an immunoprecipitation step was used to enrich tau prior to western-blotting, potentially limiting the tau fragments detected [7,33,40]. And use of postmortem CSF [27,37] may have compromised analysis as CSF tau levels are highly sensitive to the integrity of brain tissue [43].

To address these limitations, RP-HPLC was utilized to enrich and concentrate tau from a large volume of pooled, denatured CSF thereby enabling analysis of the relatively low levels of tau by western-blotting. N-terminal and mid-domain tau fragments were detected in both AD and control CSF, ranging in size from <20 kDa to ~40 kDa. In contrast, C-terminal-containing fragments were not detected using the K9JA polyclonal antibody. Furthermore, full-length tau was not detected with any of the antibodies tested. The lack of detectable C-terminal fragments was surprising given reports indicating that MTBR-containing fragments are present in CSF [22,44,45]. We confirmed the ability to fractionate and detect full-length tau and MTBR-containing fragments using purified standards (data not shown). The lack of detectable C-terminal fragments could be due to limited sensitivity of K9JA to detect these by western-blot (~30-100 pg) or the inability to resolve higher order oligomers or aggregates using the RP-HPLC system. Thus, additional enrichment methods and C-terminal-specific monoclonal antibody reagents will be needed to help resolve these issues. Nevertheless, these results demonstrate that CSF tau is composed of a complex mixture of fragments.

Tau is a putative substrate for various proteases such as calpain, caspases, cathepsins and thrombin (reviewed in 46,47). Tau fragments observed in CSF could be a direct result of processing by these proteases. Indeed, cleavages at many of the known sites may partly explain differences in absolute levels detected in the different tau ELISAs [46,47,48]. However, technical issues related to the range of tau species present and the relative affinity of antibodies for those species could also contribute to assay differences. Thus, comparisons of absolute levels between ELISAs must be interpreted with caution.

In order to investigate discriminatory potential, each ELISA was evaluated for its ability to differentiate between 20 AD and 20 matched control CSF samples. In general, all of the tau ELISAs, with the exception of HT7-77G7, behaved in a similar manner exhibiting significant (p≤0.01) differences in levels between AD and control. These findings are consistent with the high degree of correlation between assays. Despite the similarities, however, subtle differences in tau assay performance were also noted. Most interesting was the fact that the two assays specific for tau species containing N-terminal sequences, aa 9-163 (Tau12-HT7) and aa 9-198 (Tau12-BT2), exhibited the highest degree of differentiation (p values of <0.0001 and 0.0001, respectively). In comparison, differentiation was less robust (p = 0.0031) with HT7-BT2, specific for aa 159-198. This difference could be partly linked to the sensitivity of BT2 to phosphorylation at S199 [49] as levels of p199 are reported to be increased in AD CSF [11]. However BT2 sensitivity cannot account for differences between Tau12-BT2 and HT7-BT2. Taken together these findings suggest that N-terminal-containing tau species may be more sensitive biomarkers of AD. Additional N-terminal assays and larger sample cohorts will be needed to confirm these results.

Another interesting finding was the lack of a quantifiable CSF signal in the HT7-77G7 assay. This was not an assay artifact as complete recovery of a tau spike in CSF matrix was observed. Given the relatively robust signal measured in the HT7-Tau5 assay (aa 159-225), the lack of signal in the HT7-77G7 assay (aa 159-335) suggests extensive cleavage of tau between the Tau5 (aa 218-225) and 77G7 (aa 316-335) epitopes, further supporting the idea that CSF tau is fragmented. The HT7-77G7 result is also consistent with the inability to detect full-length tau by western-blotting. This was further confirmed using another ELISA, Tau12-DC39, specific for tau containing aa 9-441 [43]. As comparable to HT7-77G7, a tau signal could not be detected in CSF although a tau441 spike was readily measured (data not shown). The lack of a quantifiable HT7-77G7 signal in CSF, however, does not exclude the possibility that tau fragments containing MTBR sequences only or more C-terminal regions are present as these would be undetectable by the HT7-77G7 assay; additional C-terminal-specific assays will be needed to fully explore this idea.

Of the ptau ELISAs evaluated, the HT7-AT270 (aa 159-p181) assay exhibited the highest level of discrimination, though only slightly better than HT7-PHF6 (aa 159-p231). This finding is consistent with data reporting that p181, p231 and p199 were equivalent in their ability to discriminate AD from controls [11]. Interestingly, differentiation of AD from control was partially lost when p181 was measured in the context of tau species containing additional N-terminal sequence aa 9-p181 (Tau12-AT270). This finding is surprising given that the tau ELISAs dependent on the same N-terminal regions exhibited the most significant differences between the sample sets. These results suggest that there may be distinct pathways leading to increased CSF levels of these tau and ptau species.

In summary, our results indicate that tau is present in control and AD CSF as a mixture of fragments. Results from our novel tau and ptau assays provide evidence that the discrimination of AD from control is dependent on the subset of tau species measured and that development of more robust AD biomarkers may be possible. One limitation of the study is the relatively small sample set used. Ultimate confirmation of these findings will require analysis of larger cohorts of AD and control samples to ensure robust statistical analysis. Additional assays and reagents will also be needed to fully investigate any C-terminal fragments or aggregates present but not detected by the methods employed here. Finally, these results could have implications in the development of CSF tau and ptau biomarkers for other neurodegenerative diseases, including other tauopathies where disease-associated changes in tau and ptau have not been consistently observed.

## Methods

### Ethics statement

The study was approved by the regional ethics committee at the University of Gothenburg.

### CSF samples

Pooled control and pooled AD CSF samples were generated by board-certified laboratory technicians at the Clinical Neurochemistry Laboratory, the Sahlgrenska University Hospital in Mölndal, Sweden. Samples were categorized as control or AD on the basis of CSF tau, ptau and Aβ42 cut-points that are 90% sensitive and specific for AD (tau > 350 ng/L, ptau > 80 ng/L and Aβ42 < 530 ng/L; biomarker concentrations derived using INNOTEST ELISAs (Innogenetics, Ghent, Belgium)) [4].

Individual AD and age-matched CSF samples were purchased from Precision Med (Solana Beach, CA). Written and verbal consents were obtained from participants at screening and enrollment. For all patients, participants were > 55 years of age, in good general health having no other neurological, psychiatric or major medical diagnosis that could contribute significantly to cognitive impairment or dementia. For AD, patients were selected based upon a probable diagnosis of AD using NINCDS-ADRA criteria, a Hachinski score < (and equal to) 4 and with an MMSE between 14-26. Control subjects were classified as healthy, but no cognitive testing was performed. The complete demographic and individual data for these samples are shown in Tables S4 and S5, respectively.

### HPLC fractionation of CSF for western-blotting

Human CSF was denatured in guanidine-HCl (VWR, West Chester, PA) to a final concentration of 6 M guanidine-HCl. 24 ml injections of the denatured CSF (6 ml CSF + 18 ml guanidine-HCl) were fractionated with an Agilent 1100 series HPLC running at 1.5 ml/min over a Poros R1/10 protein column (4.6 mm X 100 mm, Applied Biosystems, Foster City, CA) heated to 65°C. 30 x 2 ml fractions were collected for each sample using an water/acetonitrile gradient (0-60% acetonitrile over 35 min) in the presence of 0.1% (volume/volume) trifluoroacetic acid. Fractions were dried to completion in a SpeedVac Explorer (Thermo Savant, Pittsburgh, PA) overnight. Dried fractions were stored at -20°C until analysis. A similar strategy was used previously to extract and enrich Aβ peptides from human plasma and CSF and to eliminate matrix interference [50,51].

### Western-blotting of fractions

Dried HPLC fractions were resuspended in tricine sample buffer, and separated on Novex 10-20% tricine gels according to manufacturer’s directions. Gels were transferred onto 0.45 µm polyvinylidene difluoride (PVDF) membrane (Invitrogen, Carlsbad, CA) in CAPS transfer buffer (pH 11.0) at room temperature for 90 min. Membranes were blocked in TBST (Tris-buffered saline with 0.1% (v/v) Tween-20) with 1% BSA (Thermo, Rockford, IL) for 1 hr. Membranes were probed with HT7 (Pierce), Tau12 (Covance, Dedham, MA), KJ9A (Dako, Carpinteria, CA), and mouse IgG1 monoclonal isotype control (Abcam, Cambridge, MA) conjugated to HRP (LYNX Rapid HRP Antibody Conjugation kit, AbD Serotec, Oxford, UK) in TBST with 1% BSA for 16 hrs at room temperature. Probed membranes were developed using SuperSignal West Femto Maximum Sensitivity Substrate (Pierce, Rockford, IL).

### INNO BIO AlzBio 3

INNO BIO AlzBio 3 was used to measure CSF Aβ_(1-42)_ (Aβ42), tau and p-tau (181) according to the manufacturer’s instructions (Innogenetics, Ghent, Belgium). Briefly, suspension array bead sets were incubated with reference standards, QC samples, human CSF along with biotinylated reporter overnight. Excess unbound material was removed via vacuum filtration, followed by incubation with streptavidin-phycoerythrin (PE) for 60 min. The antibody/peptide complexes were detected via PE fluorescence as measured by a Bio-Rad BioPlex instrument running Luminex xPonent software. Analyte levels were quantified using an unweighted 4-parameter logistic (4PL) curve fit generated from the reference standards using BioPlex manager 5.0 software

### CSF tau ELISAs

Tau ELISAs were developed using the following mouse monoclonal antibodies for capture; antibody information listed in Table 1. Tau12 (aa 9-18, SIG-39416, Covance, Princeton, NJ), HT7 (aa 159-163, MN1000, Thermo Scientific,Rockford, IL) or BT2 (aa 194-198, MN1010, Thermo Scientific,Rockford, IL). The respective analytes were detected using the following alkaline phosphatase (AP) conjugated mouse monoclonal antibodies: BT2, HT7, Tau5 (aa 218-225, SIG-39413, Covance, Princeton, NJ) or 77G7 (aa 316-335, SIG-34905, Covance, Princeton, NJ). Eptiope mapping for 77G7 is included in Supplemental methods and Figures S1 and S2. Human tau441 (tau441) recombinant protein (rPeptide, Bogart, GA) was used to generate standard curves for each of the assays. Standards were run in two-fold serial dilutions in assay buffer containing 1% BSA (w/v) and 0.05% tween-20 (v/v) in Tris buffered saline (TBS), pH 8. The tau441 standard curve range for each of the ELISAs was 400-2 pg/ml (Tau12-BT2 and HT7-Tau5), 1000-16 pg/ml (Tau12-HT7), 1000-4 pg/ml (HT7-BT2), 1000-8 pg/ml (HT7-77G7). Human CSF dilution linearity curves were run for each of the tau ELISAs with CSF at 2-fold serial dilutions from 2- to 64-fold to determine the optimal sample dilution for each of the assays. Based on the results from the CSF linearity experiment, individual CSF samples were assayed at the following dilutions in assay buffer for each of the total tau assays: 2-fold (HT7-77G7), 20-fold (Tau12-HT7), 10-fold (HT7-Tau5), 25-fold (Tau12-BT2), 30-fold (HT7-BT2). Immunodepletion and spike recovery samples were generated as described (Supplemental methods).

Tau ELISAs were run as follows. High binding black 96 well plates (Costar 3925, Corning, NY) were coated by the addition of 2.5 µg/ml (BT2, HT7) or 5 µg/ml (Tau12) capture antibodies which were diluted in Tris buffered saline (TBS), pH 8. Plate sealers were attached then the plates were incubated at 37°C for 1 hr. Plates were washed with TBST (TBS containing 0.05% Tween-20) before blocking nonspecific binding sites with 3% bovine serum albumin (BSA; protease free, fraction V; Roche Biochemicals, Indianapolis, IN) (w/v) in TBS. Plate sealers were attached and the plates were incubated at room temperature for 2-4 hrs while shaking. Plates were washed with TBST before the addition of 50 µl per well diluted human CSF and human tau441 standard curves which were each prepared in a final assay buffer concentration of 1% BSA (w/v) and 0.05% Tween-20 (v/v) in Tris buffered saline (TBS), pH 8. Plate sealers were attached, then assay plates containing human CSF and standard curves were incubated overnight at 4°C while shaking. Alkaline phosphatase (AP) conjugated BT2, HT7, Tau5 or 77G7 antibodies were diluted into assay buffer before being added to the assay plate (50 µl per well) to co-incubate with human CSF and htau441 standard curves for 1 hr at room temperature while shaking. Plates were washed with TBST before being developed using alkaline phosphatase substrate (T2214; Applied Biosystems, Foster City, CA). Luminescence counts were measured using a Packard TopCount (PerkinElmer, MA). Log-transformed luminescence counts from individual samples were interpolated to concentration using a second-order polynomial fit to the respective standards (GraphPad Prism 5.00, GraphPad Software, San Diego, CA). CSF tau levels were plotted after correction for dilution factor in the respective assays. Assay lower limit of quantitation (LLQ) was set based on the lowest calibrator demonstrating acceptable total error (bias + precision of < 30%).

### Human Tau441 Spike Recovery in HT7-77G7 ELISA

A pooled CSF sample from AD patients and an age-matched pooled control CSF sample were 2-fold serially diluted from 2- to 256-fold in a final assay buffer concentration of 1% BSA (w/v) and 0.05% tween-20 (v/v) in Tris buffered saline (TBS), pH 8 before an aliquot of each was spiked with recombinant human tau441 protein (rPeptide, Bogart, GA) at a final concentration of 100 pg/ml. Diluted CSF samples with and without tau441 spike were assayed in the HT7-77G7 ELISA as described above.

### CSF ptau ELISAs

Human CSF was analyzed in three different ptau assays: HT7-AT270 (p181) and HT7-PHF6 (p231) and Tau12-AT270 (p181). The HT7-AT270 and HT7-PHF6 ELISAs utilized HT7 (amino acids 159-163), while the Tau12-AT270 assay used Tau12 (amino acids 9-18), as the capture antibody, and the respective analytes were detected using alkaline phosphatase (AP) conjugated AT270 pT181 tau antibody (MN1050, Thermo, Rockford, IL) or PHF6 pT231 specific monoclonal antibody (SIG-39430, Covance, Dedham, MA); antibody information listed in Table 1. Standards for the 3 different assays were custom synthesized (Abgent Inc., San Diego, CA) and contained sequences with the respective capture and detection epitopes as follows: the HT7-AT270 and HT7-PHF6 assay standards used native human tau sequence of aa 155–207 and aa 155–236, respectively, with the Thr181 and Thr 231 residues being phosphorylated; the Tau12-AT270 assay standard consisted of aa 5-28 linked with a polyethylene glycol (PEG12) linker to aa 174-187, with a phosphorylated Thr181 residue. Standard purity was verified at the Keck Biotechnology Resource Laboratory at Yale University. Standards were run in 2-fold serial dilution in assay buffer containing 0.3% BSA in PBS with 0.05% Tween, with a range of 125 to 1 pg/ml for HT7-AT270, and 500 to 4 pg/ml for HT7-PHF6 and Tau12-AT270 tau assays. CSF samples were run neat for the HT7+pT231 tau assay, while the Tau12+pT181 and HT7+pT181 tau assays were run at 2- and 4-fold dilution in assay buffer. Immunodepletion and spike recovery samples were generated as described (Supplemental methods).

pTau ELISAs were run as follows. Black high-binding plates (Costar, Corning, NY) were coated with 2.5 µg/ml of capture antibodies HT7 or Tau12 in carbonate-bicarbonate buffer at pH 9.4 (Thermo, Rockford, IL). After overnight incubation at 4°C, plates were washed with PBS and nonspecific binding sites were blocked using 3% BSA in PBS buffer for at least 4 hrs at 4°C. Standards and CSF samples (50 µL) were added to plates, followed by 50 µL of AP conjugated AT270 (pThr181) and PHF6 (pThr231) tau antibodies for the respective assays. After overnight incubation at 4°C, plates were washed with PBS (containing 0.05% tween) and developed using alkaline phosphatase substrate (T2214; Applied Biosystems, Foster City, CA). Luminescence counts were measured using Envision (PerkinElmer, MA). Log-transformed luminescence counts from individual samples were interpolated to concentration using a 3rd order polynomial fit to the respective standards (GraphPad Prism 5.00, GraphPad Software, San Diego CA) CSF ptau levels were plotted after correction for dilution factor in the respective assays. Assay lower limit of quantitation (LLQ) was set based on the lowest calibrator demonstrating acceptable total error (bias + precision of < 30%).

### Statistics

Statistical calculations were performed using GraphPad Prism 5.00 (GraphPad Software, San Diego, CA). Differences in biomarker levels between AD and control samples were examined using unpaired two-tailed Student’s t-test. Data was log-transformed prior to t-test comparisons to correct for non-Gaussian distributions as determined by the D’Agostino & Pearson normality test. Results were considered significant for p-values < 0.01

## Supporting Information

Figure S1 **Tau peptides used for 77G7 antibody epitope mapping.** A set of 29 overlapping peptides spanning the length of human tau 441 were generated, coupled to beads and used to map the epitope of tau antibody 77G7 using a Luminex-based multiplex assay.
(DOCX)

Figure S2 **Mapping the tau epitope of antibody 77G7.** Left panel) 77G7 exhibited binding to human Tau 441 and the C-terminal human tau fragment aa 231-441 but not to the tau fragments aa 1-125 or aa 126-230; 77G7 also bound to the other tau isoforms (data not shown). Mid-domain tau antibody HT7 exhibited binding to tau 441 and fragment aa 126-230 as expected. Binding was not observed with the anti-IL6 control antibody. Right panel) A set of 29 overlapping peptides spanning the length of human tau 441 were generated, coupled to beads and used in a Luminex-based multiplex assay to screen 77G7, HT7 and anti-IL6 control. 77G7 exhibited binding to peptide 22 (aa 316-335) and much lower level binding to peptide 25 but none of the other peptides (right panel and data not shown). HT7 exhibited binding to peptide 11 (aa 150-170) but none of the other peptides (right panel and data not shown) as expected while the control anti-IL6 antibody did not react with any of the peptides tested.
(DOCX)

Figure S3 **Verification of signal specificity in tau ELISAs by immunodepletion.** Pooled CSF was immunodepleted with tau antibody HT7 (IP) or treated with protein A/G beads alone (control). Samples were analyzed in tau ELISAs A) HT7-BT2, B) HT7-Tau5, C) Tau12-BT2 and D) Tau12-HT7. Data represents mean ± SEM from 3-4 determinations. Dashed lines indicate the assay LLQ corrected for CSF dilution.
(DOCX)

Figure S4 **Spike recovery in tau ELISAs.** Pooled CSF samples were treated with tau 441 spikes ranging from 10-800 pg/ml. Spiked samples and a matching untreated control were analyzed in tau ELISAs A) HT7-BT2, B) HT7-Tau5, C) Tau12-BT2 and D) Tau12-HT7 and spike recovery determined (%). Data represents mean ± SEM from 3 determinations. Dashed lines indicate 100% spike recovery.
(DOCX)

Figure S5 **Verification of signal specificity in ptau ELISAs by immunodepletion and peptide competition.** Pooled CSF samples from healthy control subjects (black bars) or AD patients (red bars) were immunodepleted with tau antibody HT7 (IP) or protein A/G beads alone (control). CSF samples were also treated with pT181 or pT231 peptides for competition analysis. Samples were analyzed in ptau ELISAs A) HT7-AT270, B) HT7-PHF6, and C) Tau12-AT270. Data represents mean ± SEM from 3 determinations. Dashed lines indicate the assay LLQ corrected for CSF dilution.
(DOCX)

Figure S6 **Spike recovery in ptau ELISAs.** Pooled CSF samples were treated with pT181 or pT231 spikes ranging from 12.5-200 pg/ml. Spiked samples and a matching untreated controls were analyzed in ptau ELISAs A) HT7-AT270, B) HT7-PHF6, and C) Tau12-AT270 and spike recovery determined (%). Data represents mean ± SEM from 3 determinations. Dashed lines indicate 100% spike recovery.
(DOCX)

Figure S7 **Analysis of tau and ptau levels in 20 AD and 20 control CSF samples.** A set of 20 AD and 20 age-matched normal control CSF samples were analyzed using INNO-BIA AlzBio3. Statistics based on 2-tailed Student’s t test comparison of log-transformed data (tau and ptau) or untransformed data (Aβ42). * p < 0.05; ** p< 0.01; *** p < 0.001.
(DOCX)

Methods S1 **Epitope mapping and immunodepletion and spike recovery.**
(DOCX)

Table S1 **Analysis of tau assay ratios in 20x20 sample set.**
(DOCX)

Table S2 **Tau ELISA correlations.**
(DOCX)

Table S3 **Analysis of ptau assay ratios in 20 x 20 sample set.**
(DOCX)

Table S4 **Complete demographic information for 20 x 20 sample set.**
(DOCX)

Table S5 **Individual data for 20 x 20 sample set.**
(DOCX)
