# Supplementary material for: Characterization of Novel CSF Tau and ptau Biomarkers for Alzheimer’s Disease
Source: PLoS One. 2013 Oct 7;8(10):e76523. doi: 10.1371/journal.pone.0076523 (PMC3792042; doi:10.1371/journal.pone.0076523)
Supplement: Figure S1 — Tau peptides used for 77G7 antibody epitope mapping. A set of 29 overlapping peptides spanning the length of human tau 441 were generated, coupled to beads and used to map the epitope of tau antibody 77G7 using a Luminex-based multiplex assay. (DOCX) [file pone.0076523.s001.docx]

### Figure S1
