# Supplementary material for: Characterization of Novel CSF Tau and ptau Biomarkers for Alzheimer’s Disease
Source: PLoS One. 2013 Oct 7;8(10):e76523. doi: 10.1371/journal.pone.0076523 (PMC3792042; doi:10.1371/journal.pone.0076523)
Supplement: Figure S2 — Mapping the tau epitope of antibody 77G7. Left panel) 77G7 exhibited binding to human Tau 441 and the C-terminal human tau fragment aa 231-441 but not to the tau fragments aa 1-125 or aa 126-230; 77G7 also bound to the other tau isoforms (data not shown). Mid-domain tau antibody HT7 exhibited binding to tau 441 and fragment aa 126-230 as expected. Binding was not observed with the anti-IL6 control antibody. Right panel) A set of 29 overlapping peptides spanning the length of human tau 441 were generated, coupled to beads and used in a Luminex-based multiplex assay to screen 77G7, HT7 and anti-IL6 control. 77G7 exhibited binding to peptide 22 (aa 316-335) and much lower level binding to peptide 25 but none of the other peptides (right panel and data not shown). HT7 exhibited binding to peptide 11 (aa 150-170) but none of the other peptides (right panel and data not shown) as expected while the control anti-IL6 antibody did not react with any of the peptides tested. (DOCX) [file pone.0076523.s002.docx]

### Figure S2
