# Supplementary material for: Characterization of Novel CSF Tau and ptau Biomarkers for Alzheimer’s Disease
Source: PLoS One. 2013 Oct 7;8(10):e76523. doi: 10.1371/journal.pone.0076523 (PMC3792042; doi:10.1371/journal.pone.0076523)
Supplement: Figure S3 — Verification of signal specificity in tau ELISAs by immunodepletion. Pooled CSF was immunodepleted with tau antibody HT7 (IP) or treated with protein A/G beads alone (control). Samples were analyzed in tau ELISAs A) HT7-BT2, B) HT7-Tau5, C) Tau12-BT2 and D) Tau12-HT7. Data represents mean ± SEM from 3-4 determinations. Dashed lines indicate the assay LLQ corrected for CSF dilution. (DOCX) [file pone.0076523.s003.docx]

### Figure S3
