# Supplementary material for: Characterization of Novel CSF Tau and ptau Biomarkers for Alzheimer’s Disease
Source: PLoS One. 2013 Oct 7;8(10):e76523. doi: 10.1371/journal.pone.0076523 (PMC3792042; doi:10.1371/journal.pone.0076523)
Supplement: Figure S4 — Spike recovery in tau ELISAs. Pooled CSF samples were treated with tau 441 spikes ranging from 10-800 pg/ml. Spiked samples and a matching untreated control were analyzed in tau ELISAs A) HT7-BT2, B) HT7-Tau5, C) Tau12-BT2 and D) Tau12-HT7 and spike recovery determined (%). Data represents mean ± SEM from 3 determinations. Dashed lines indicate 100% spike recovery. (DOCX) [file pone.0076523.s004.docx]

### Figure S4
