# Supplementary material for: Characterization of Novel CSF Tau and ptau Biomarkers for Alzheimer’s Disease
Source: PLoS One. 2013 Oct 7;8(10):e76523. doi: 10.1371/journal.pone.0076523 (PMC3792042; doi:10.1371/journal.pone.0076523)
Supplement: Figure S5 — Verification of signal specificity in ptau ELISAs by immunodepletion and peptide competition. Pooled CSF samples from healthy control subjects (black bars) or AD patients (red bars) were immunodepleted with tau antibody HT7 (IP) or protein A/G beads alone (control). CSF samples were also treated with pT181 or pT231 peptides for competition analysis. Samples were analyzed in ptau ELISAs A) HT7-AT270, B) HT7-PHF6, and C) Tau12-AT270. Data represents mean ± SEM from 3 determinations. Dashed lines indicate the assay LLQ corrected for CSF dilution. (DOCX) [file pone.0076523.s005.docx]

### Figure S5
