# Supplementary material for: Characterization of Novel CSF Tau and ptau Biomarkers for Alzheimer’s Disease
Source: PLoS One. 2013 Oct 7;8(10):e76523. doi: 10.1371/journal.pone.0076523 (PMC3792042; doi:10.1371/journal.pone.0076523)
Supplement: Figure S6 — Spike recovery in ptau ELISAs. Pooled CSF samples were treated with pT181 or pT231 spikes ranging from 12.5-200 pg/ml. Spiked samples and a matching untreated controls were analyzed in ptau ELISAs A) HT7-AT270, B) HT7-PHF6, and C) Tau12-AT270 and spike recovery determined (%). Data represents mean ± SEM from 3 determinations. Dashed lines indicate 100% spike recovery. (DOCX) [file pone.0076523.s006.docx]

### Figure S6
