# Supplementary material for: Characterization of Novel CSF Tau and ptau Biomarkers for Alzheimer’s Disease
Source: PLoS One. 2013 Oct 7;8(10):e76523. doi: 10.1371/journal.pone.0076523 (PMC3792042; doi:10.1371/journal.pone.0076523)
Supplement: Figure S7 — Analysis of tau and ptau levels in 20 AD and 20 control CSF samples. A set of 20 AD and 20 age-matched normal control CSF samples were analyzed using INNO-BIA AlzBio3. Statistics based on 2-tailed Student’s t test comparison of log-transformed data (tau and ptau) or untransformed data (Aβ42). * p < 0.05; ** p< 0.01; *** p < 0.001. (DOCX) [file pone.0076523.s007.docx]

### Figure S7
