# Supplementary material for: Characterization of Novel CSF Tau and ptau Biomarkers for Alzheimer’s Disease
Source: PLoS One. 2013 Oct 7;8(10):e76523. doi: 10.1371/journal.pone.0076523 (PMC3792042; doi:10.1371/journal.pone.0076523)
Supplement: Methods S1 — Epitope mapping and immunodepletion and spike recovery. (DOCX) [file pone.0076523.s008.docx]

## Supplemental methods

### Epitope mapping

A multiplexed immunoassay was developed on the Luminex platform for mapping tau antibody epitopes The assay consisted of 41 different antigens, each covalently coupled to a unique Luminex bead set using standard amine coupling protocols. These antigens included all six human tau isoforms (rPeptide, Bogart, GA), a set of 29 synthetic overlapping tau sequence peptides (GenScript, Piscataway NJ) spanning the length of human tau 441 (Supplemental figure 1), and 3 tau fragments (aa 1-125, 126-230, 231-441) generated using standard in vitro transcription/translation system. Beads were conjugated using a two-step carbodiimide procedure. Briefly, 1 mL of beads (1.25 x 107/mL) was pelleted by centrifugation for 1 min at 10000 x g at 4° C in an Eppendorf 5415D centrifuge (Westbury, NY). Beads were washed and resuspended in 500 μL of 0.1M sodium phosphate buffer pH 4.8 (activation buffer) followed by 15 seconds of vortexing and 15 seconds of sonication. Beads were washed 2x times with activation buffer, resuspended in 200 μL of freshly prepared 5 mg/mL of EDC (1-Ethyl-3-[3-dimethylaminopropyl]carbodiimide hydrochloride, inactivation buffer), and incubated in a rotator for 20 min at RT protected from light. Beads were then washed and resuspended in 500 μL of antigen in PBS(100 μg for the Tau isoforms and 20 μg for the peptides) and incubated for 2hrs at RT in a rotator protected from light. Beads were then washed and incubated with 0.5 mL of blocking buffer (PBS, 1% (w/v) BSA, 0.02% (w/v) Tween-20) in a rotator for 1-hour at RT protected from light. Finally, the beads were counted with a hemacytometer and resuspended in blocking buffer at 2 x 106 beads/mL. Beads were stored protected from light at 4° C. Prior to testing, the bead sets were mixed together to form a suspension array. Antibodies for epitope mapping were incubated with the bead mix, washed, and subsequently incubated with PE-labeled anti-mouse IgG (H+L) reporter antibodies. The beads were then analyzed on a Bioplex Luminex 100 instrument (Bio-Rad Laboratories, Hercules, CA)

### Immunodepletion and spike recovery

Human control CSF was diluted 2-fold into assay buffer (final concentration of 1% BSA in TBS with 0.05% Tween-20) before an aliquot was removed and HT7 was added to a final concentration of 1µg/ml. CSF and HT7 were co-incubated for 1 hour at 4 degrees before being added to protein A/G agarose (Thermo Fisher Scientific, Rockford, IL) at a 9:1 ratio. The protein A/G beads were blocked with 10 volumes of 2% BSA (w/v )in TBS for 1 hour before use. The remaining diluted CSF sample that was not immunodepleted with HT7 was incubated with protein A/G beads to serve as a control. CSF and protein A/G beads were incubated overnight at 4 degrees C. Beads were spun out before the supernatant was collected, diluted and assayed in CSF tau ELISAs. CSF was assayed at a 3-fold (HT7+Tau5), 5-fold (Tau12+BT2 and Tau12+HT7) or 10-fold dilution (BT2+HT7).

For ptau ELISAs, pooled CSF samples from healthy controls and AD subjects were immunodepleted with tau antibody HT7 conjugated to CNBr activated Sepharose 4B beads or unconjugated inactive sepharose 4B beads (GE Healthcare, Pittsburgh, PA) following overnight incubation at 4C in a rotating holder. Next day, CSF samples were spun at 1000 rpm for 5 min and supernatants (depleted CSF) were run in respective ptau assays. For phospho-peptide depletion studies, CSF samples were spiked with 100 ng/ml of pT181 (PPAPK-T(PO4)-PP) or pT231 (KVAVVR-T(PO4)-PPK) peptides and run in respective ptau assays (HT7-AT270, HT7-PHF6, and Tau12-AT270). For spike-recovery studies, control CSF samples diluted in 0.3% BSA/PBS were spiked with the respective ptau standards over a range of concentrations and run in ELISA’s along with non-spiked samples. Spike recovery was estimated as a percent recovery of signal above basal signal levels in non-spiked CSF samples.
