# Supplementary material for: Characterization of Novel CSF Tau and ptau Biomarkers for Alzheimer’s Disease
Source: PLoS One. 2013 Oct 7;8(10):e76523. doi: 10.1371/journal.pone.0076523 (PMC3792042; doi:10.1371/journal.pone.0076523)
Supplement: Table S5 — Individual data for 20 x 20 sample set. (DOCX) [file pone.0076523.s013.docx]

### Table S5 Individual data for 20 x 20 sample set
